# Symptoms characteristics of personality disorders associated with suicidal ideation and behaviors in a clinical sample of adolescents with a depressive disorder

**DOI:** 10.3389/fpsyt.2023.1269744

**Published:** 2023-12-11

**Authors:** Anthony Joseph Gifuni, Michel Spodenkiewicz, Geneviève Laurent, Sasha MacNeil, Fabrice Jollant, Johanne Renaud

**Affiliations:** ^1^McGill Group for Suicide Studies, Douglas Mental Health University Institute, Montreal, QC, Canada; ^2^Department of Psychiatry, Faculty of Medicine and Health Sciences, McGill University, Montreal, QC, Canada; ^3^INSERM UMR-1178, Moods Team, CESP, Le Kremlin-Bicêtre, France; ^4^Department of Psychology, Concordia University, Montreal, QC, Canada; ^5^Faculty of Medicine and Health Sciences, McGill University, Montreal, QC, Canada; ^6^Division of Child Psychiatry, Department of Psychiatry, Faculty of Medicine and Health Sciences, McGill University, Montreal, QC, Canada

**Keywords:** symptoms characteristics of personality disorders, suicidal ideation and behaviors, depressive disorder, adolescence, suicide attempt

## Abstract

**Introduction:**

Pathological personality traits have repeatedly been identified as important risk factors for suicidal ideation and behaviors. Moreover, impulsive-aggressive traits, have shown a consistent association with suicidal behaviors across the lifespan. Adolescence represents a critical period for the emergence of different personality traits, mood disorders, and suicidal behaviors, but the relationship between these variables remain poorly understood.

**Methods:**

These variables were examined in a cross-sectional case–control design involving three groups: 30 adolescents with a depressive disorder and past suicide attempt (Mean Age = 16.2, Females = 26), 38 adolescents with a depressive disorder but without past suicide attempt (Mean age = 16.0, Females = 29), and 34 healthy adolescent controls (Mean age = 15.2, Females = 22). Suicidal ideations were indexed using Suicidal Behavior Questionnaire (SBQ-R), psychiatric disorder assessed using a semi-structured questionnaire (K-SADS-PL), depressive symptoms with the Beck Depressive Inventory (BDI), symptoms characteristics of personality disorders with the Scheduled Clinical Interview for the DSM-IV (SCID-II) screening questionnaire, and impulsivity with the Barratt Impulsivesness Scale (BIS).

**Results:**

Findings showed that impulsivity (*F* = 11.0, *p* < 0.0001) and antisocial symptoms characteristics of personality disorders (*p* < 0.001, d = 0.70) displayed the most robust association with adolescent suicide attempts. Borderline symptoms characteristics of personality disorders did not discriminate attempters from non-attempters but presented high correlations with suicidal ideation and depression severity. In an item-wise analysis, suicide attempt status was uniquely correlated with symptoms characteristics of an antisocial personality disorder. Suicide attempt status also correlated with non-suicidal self-injury and a chronic feeling of emptiness.

**Discussion:**

The caveats of this cross-sectional study include the stability of symptoms characteristics of personality disorders in adolescence and the limited sample size. In sum, suicidal behaviors were characteristically correlated with increased impulsivity and antisocial symptoms characteristics of personality disorders, but other symptoms characteristics of personality disorders were relevant to adolescent depression and suicidal ideation. Understanding the emergence of symptoms characteristics of personality disorders and suicidal behaviors in a developmental context can ultimately inform not only the neurobiological origin of suicidal behaviors, but also provide new avenues for early detection and intervention.

## Introduction

Adolescent suicidal behaviors are major public health concerns in our society (1–3). Suicide represents the fourth cause of mortality in youth aged 15–29 years across the world (4). In Quebec, lifetime prevalences of passive suicidal ideation, serious suicidal ideation, and suicide attempt in adolescents were 22.2, 9.8, and 6.7%, respectively. While rare in the first decade of life, suicidal thoughts and behaviors emerge in adolescence (2, 5). In addition to psychiatric illness, socio-environmental stress such as academic difficulties, parent–child conflict, bullying, gender inequality or other forms of interpersonal violence, are often associated with adolescent suicidal behaviors (6, 7). While psychiatric disorders and external stress are often correlated retrospectively with past suicide attempt (8–10), their presence has limited capacity to predict suicidal behaviors even while applying advanced suicide prediction models (11). This observation highlights the role of individual dispositional factors in mediating the relationship between stress and suicidal behaviors (12).

Among the various psychological factors moderating suicide risk, several symptoms characteristics of personality disorders (SCPD) show consistent associations with suicidal thoughts and behaviors (13–15). In adults and adolescents, several differences in SCPD have been found in individuals with suicidal thoughts or behaviors compared to the ones without these pathological traits such as higher levels of neuroticism (7, 16–18), lower levels of extroversion (17), increased perfectionism (15, 19) and more impulsivity (20, 21). SCPD predisposing to suicidal ideation might differ from those predisposing to suicidal attempts (22, 23). Furthermore, evidence demonstrates that an important subset of suicide attempters displays a combination of higher levels of impulsivity and a greater tendency for aggression (24, 25). In particular, more violent means for suicide were associated with a personality characterized by impulsive-aggression (26). Thus, individual differences in personality, which involves the complex integration of emotional regulation, cognition and interpersonal skills, play an important role in the suicidal diathesis.

Examining the relationship of personality traits in adolescence and the suicidal diathesis has important clinical implications. Firstly, adolescence is often conceptualized as the developmental period when personality disorders start (27). SCPD emerging in adolescence often persist into adulthood (28). Secondly, SCPD are related to the risk of depressive disorders, anxiety disorders and substance use disorder (29, 30) which all contribute to greater suicide risk. Finally, adolescent-emergent personality disorders can be malleable therapeutic targets for the management of suicidal behaviors (31). Although personality is are progressively consolidated during adolescence and are associated with suicide risk, the relationship of SCPD with adolescent suicidality remains poorly understood since previous studies concerned an adult population (32). The role of the current study was therefore to shed new light on the association of SCPD with suicidal behaviors in adolescents with depressive pathologies. The study was conducted with the hypothesis that adolescents with past suicidal behaviors would present an impulsive-aggressive phenotype.

## Methods

### Participants

A total of 102 adolescents, aged between 11 and 17 years old, were recruited to form three groups: (1) Adolescents with a depressive disorder and a history of at least one suicide attempt (SA; *n* = 30), (2); adolescents with a depressive disorder without history of suicide attempt (i.e., patient controls; PC; *n* = 38), (3); and adolescents without a history of psychiatric disorder or suicide attempt (i.e., healthy controls; HC; *n* = 34). Suicide attempts were defined in accordance with the Columbia Classification Algorithm of Suicide Assessment (33), i.e., a self-injurious behavior perpetrated with intent to die. Therefore, aborted and interrupted suicide attempts (attempts halted by the participant or another person before any potentially self-harm occur) or patients with exclusively non-suicidal self-injuries were not included in the SA group. Depressive disorders included major depressive disorders, dysthymia, and depressive disorder not otherwise specified. Exclusion criteria comprised neurological disorders (e.g., epilepsy, brain tumor), traumatic brain injury (>1 min unconsciousness, neuroimaging anomaly, persistent post-concussive symptoms), autism spectrum disorder, bipolar disorders, psychotic disorders, intelligence quotient (IQ) less than 70, and pregnancy. This sample has been described in a previous article (34). Given evidence of heritable phenotypes associated with suicide attempts (35, 36), a family history of suicide attempts was an additional exclusion criterion for the HC group.

Recruitment of patients (SA and PC group) took place from September 2012 to January 2019, primarily at the Depressive Disorders Clinic for adolescents at the Douglas Institute, Montréal (Quebec, Canada), with additional participants recruited from existing studies or referred from child psychiatrists working at affiliated community clinics (CLSCs). HC were recruited from the community through advertisements posted in schools, local clinics and hospitals, youth centers, and groups of parents on social media. The HC group of participants was added to the initial protocol in 2017, with the objective of comparing both patient groups to typically developing youth. Participants were initially screened with a phone interview by a research assistant. All participants were compensated monetarily, and consent was obtained both from adolescents and a least one of their parents or legal guardians. The research protocol was approved by the Douglas Institute Research Ethics Board.

### Clinical measures

All participants were assessed by the primary investigator and child and adolescent psychiatrist (JR) in semi-structured interviews using the K-SADS-PL (37) to assign psychiatric diagnoses based on DSM-IV classification of mental disorders. Information regarding the suicide attempt history was assessed with the Suicide History Questionnaire (SHQ) (in house questionnaire) cross-validated with notes from the patient’s medical file (reviewed by AJG), and information from the clinical interview. Suicidal ideation was acknowledged as the total score on the Suicidal Behavior Questionnaire (38), an easily administered 4-item questionnaire assessing the frequency and intensity of suicidal ideation and the self-reported probability of future suicide attempts. Participants also completed the self-administered SCID-II screening questionnaire to provide information on the lifetime SCPD and were assessed by a trained clinician (39). In the current study, the item assessing history of suicide attempts (part of symptoms characteristics of a borderline personality disorder) was removed from analysis, as it was directly related to group identification and would bias analysis. The presence of lifetime non-suicidal self-injury (NSSI) was tracked with item 98 of the SCID-II (“*Have you ever cut, burned, or scratched yourself on purpose?*”). To facilitate comparisons among SCPD, conduct disorder symptoms were examined as symptoms characteristics of an antisocial personality disorder since these items overlap within the Kiddie Schedule for Affective Disorders and Schizophrenia (K-SADS) (for conduct disorder) and the SCID-II (for antisocial personality disorder), and are considered as a ‘circular issue’ within the psychiatric classifications (40)’. Self-reported impulsivity was measured with the Barratt Impulsiveness Scale (BIS) (41). Depressive symptoms and hopelessness were assessed, respectively, with the Beck Depression Inventory (BDI-II) (42) and Beck Hopelessness Scale (BHS) (43, 44). Intelligence (with subscales for working memory, perceptive reasoning, verbal comprehension and processing speed) was measured with the Wechsler Intelligence Scale for Children (4th edition) (45) and with the Wechsler Adult Intelligence Scale (WAIS) for participants aged 17 and 18 (46).

### Statistical analyses

The distribution of quantitative variables was first checked for normality with the Kolmogorov–Smirnov and the Shapiro–Wilk test. Socio-demographic and clinical characteristics were compared across all three groups using one-way analysis of variance (ANOVA) for continuous variables and chi-square tests for discrete variables. A statistical threshold with value of p of 0.05 was set *a priori* for all analysis. SCPD scores collected with the SCID-II were aggregated for each personality disorder, with higher scores reflecting the endorsement of more traits within each personality disorder category. These continuous scores of SCPD for all personality categories were residualized for age, sex, and IQ before group comparison. In significant three-way ANOVAs, Tukey post-hoc tests were conducted for pairwise comparisons.

Sensitivity analysis was conducted to account for the effect of psychiatric diagnosis or medication status that were statistically different between groups. The correlations between clinical variables (including suicidal ideation) and SCID-II SCPD scores in each diagnostic category were cross-correlated into a correlational matrix. For a finer-grain analysis of the association of each SCPD with suicidality, we conducted two correlational analyses between all SCID-II items with (1) history of suicide attempt and (2) suicidal ideation score from the SBQ. For history of suicide attempt (dichotomous variable), we calculated correlations with mean square contingency coefficient (r_phi_) and for suicidal ideation with the point biserial correlation coefficient (r_pb_). Correlation coefficients were then ranked to determine which category of SCPD scores correlated the most with suicidal ideation or attempt. All statistical analyses and statistical visualizations were conducted with R, implemented in RStudio (version 1.1.383).

## Results

### Socio-demographic and clinical characteristics of the sample

The socio-demographic and clinical characteristics of the sample are presented in Tables 1, 2. The SA and PC group were comparable in terms of sex and age, while the HC group was slightly younger (15 versus 16 years old). IQ was significantly different across groups, with an overall IQ score significantly lower in SA versus HC. The only IQ subscale difference across groups was verbal comprehension. Depression and hopelessness scores, measured, respectively, using the BDI and the BHS were markedly higher in SA and PC versus HC, with no difference between SA and PC. The SBQ-R (used for indexing suicidal ideation) scores were significantly different across all groups, with significantly higher scores in SA versus PC, and, in turn, in PC versus HC (see Table 1).

**Table 1:** Sociodemographic and clinical features of the three adolescent groups.

	SA (*N* = 30)		PC (*N* = 38)		HC (*N* = 34)		Group analysis ANOVA/Chi-square	Post-Hoc Testing
	N/Mean	*%/SD*	N/Mean	%/SD	N/Mean	*%/SD*		
*Sex (Females)*	26	87	29	76	22	65	X = 4.51, *p* = 0.11	–
*Age (yrs)*	16.2	1.0	16.0	1.5	15.2	1.5	F = 4.90, *p* = 0.0094	SA = PC > HC
*Ethnicity*								
Asian	1	3.3	2	5.3	2	6.1	X = 0.26, *p* = 0.88	–
Black	1	3.3	1	2.6	5	15.2	X = 5.15, *p* = 0.08	–
Caucasian	23	76.7	29	76.3	22	66.7	X = 1.09, *p* = 0.58	–
First Nation	2	6.1	6	15.8	2	6.7	X = 2.37, *p* = 0.31	–
Hispanic	2	6.7	1	2.6	0	0.0	X = 2.45, *p* = 0.29	–
Multiethnic	0	0.0	1	2.6	2	6.1	X = 2.03, *p* = 0.36	–
*Parental education (highest achieved)*								
Elementary school	8	26.7	3	7.9	0	0.0	X = 12.1, *p* = 0.0023	SA = PC > HC
High school	5	16.7	8	21.1	8	24.2	X = 0.55, *p* = 0.76	–
College	4	13.3	3	7.9	7	21.1	X = 2.63, *p* = 0.27	–
University	13	43.3	24	63.2	18	54.5	X = 2.66, *p* = 0.26	–
*Living situation*								
Both parents	11	36.7	18	47.4	22	66.7	X = 5.90, *p* = 0.052	–
A parent and a partner	8	26.7	6	15.8	3	9.1	X = 0.95, *p* = 0.62	–
A parent or a step-parent	7	23.3	9	23.7	7	21.2	X = 0.38, *p* = 0.83	–
Alone or with roommates	1	3.3	0	0.0	0	0.0	X = 0.03, *p* = 0.98	–
Other (e.g., Youth residence)	3	10.0	5	13.2	1	3.0	X = 3.95, *p* = 0.14	–
*BDI*	30.2	12.5	26.4	12.7	5.7	5.7	F = 49.77, *p* < 0.0001	SA = PC > HC
*BHS*	11.1	5.5	9.5	4.5	3.3	2.9	F = 29.16, *p* < 0.0001	SA = PC > HC
*SBQ-R*	14.0	2.3	10.8	3.6	3.9	1.4	F = 121.8, *p* < 0.0001	SA > PC > HC
*BIS*								
Attentional	20.5	3.3	17.5	3.4	14.8	4.2	F = 17.0, *p* < 0.0001	SA > PC > HC
Motor	21.5	4.5	19.7	4.3	18.7	3.8	F = 3.22, *p* < 0.05	SA > HC
Non-planning	30.4	3.5	28.6	5.5	25.8	5.8	F = 6.03, *p* < 0.01	SA = PC > HC
Total	72.5	8.9	65.7	11.2	59.3	11.1	F = 11.0, *p* < 0.0001	SA > PC > HC
*Full scale IQ*								
Verbal comprehension	32.1	8.6	34.5	8.1	37.4	5.7	F = 3.35, *p* < 0.05	HC > SA
Perceptual reasoning	31.2	8.3	32.7	6.2	32.4	5.9	F = 0.33, *p* > 0.05	–
Working memory	19.9	4.0	22.0	5.3	22.5	5.3	F = 1.9, *p* > 0.05	–
Processing speed	18.6	3.9	20.0	6.0	21.8	5.3	F = 2.24, *p* > 0.05	-
Total	100.9	15.5	106.7	14.0	110.7	12.6	F = 3.14, *p* < 0.05	HC > SA

BDI, Beck Depression Inventory, BIS, Barratt Impulsiveness Scale, BHS, Beck Hopelessness Scale, SBQ-R, Suicidal Behavior Questionnaire – Revised, HC, Healthy Controls, PC, Psychiatric Controls, SA, Suicide Attempters.

**Table 2:** Psychiatric diagnosis, medication and characteristic feature of depressed adolescents with past suicide attempt (SA) and without past suicide attempt (PC).

	SA (N = 30)		PC (N = 38)		
	N/Mean	*%/SD*	N/Mean	%/SD	*Chi-square (X, p)*
*Psychiatric diagnosis*					
MDD	15	50.0	29	76.3	4.00, *p* = 0.046^*^
Dysthymia	5	16.7	7	18.4	0.0, *p* = 1.0
DD-NOS	11	36.7	4	10.5	5.22, *p* = 0.02^*^
Anxiety disorder	12	40.0	21	55.3	1.01, *p* = 0.31
PTSD	0	0.0	3	7.9	0.96, *p* = 0.33
Eating Disorder	3	10.0	4	10.5	0.00, *p* = 1.0
ADHD	9	30.0	3	7.9	4.21, *p* = 0.04^*^
ODD	1	3.3	0	0.0	0.014, *p* = 0.9
Substance use disorder	4	13.3	0	0.0	3.24, *p* = 0.07
*Medication*					
Antidepressant	15	50.0	24	55.8	0.16, *p* = 0.68
Mood stabilizer	2	6.7	2	4.7	0.04, *p* = 0.83
Low-dose antipsychotic	11	36.7	8	21.1	1.32, *p* = 0.25
Regular dose antipsychotic	1	3.3	2	5.3	1.17, *p* = 1.0
Benzodiazepine	1	3.3	1	3.3	0.00, *p* = 1.0
Stimulant	9	30.0	2	4.7	5.85, *p* = 0.01^*^
*Lifetime history of NSSI*	26	86.7	25	65.8	2.86, *p* = 0.09
*Age at first SA (yrs)*	14.28	1.28	–	–	–
*Range of age at first SA (min-max, yrs)*	10.61–16.61		–	–	–
*Recency of last SA (Months)*	13.83	11.9	–	–	–

ADHD, Attention Deficit Hyperactivity Disorder, DD-NOS, Depressive Disorder Not Otherwise Specified, MDD, Major Depressive Disorder, NSSI, Non Suicidal Self-Injury, ODD, Oppositional Defiant Disorder, SA, Suicide Attempters, PC, Psychiatric Controls, PTSD, Post-Traumatic Stress Disorder.

The most common diagnosis in patient groups was major depressive disorder (MDD). However, a higher proportion of SA had depressive disorders not otherwise specified (DD-NOS). Other comorbidities (Anxious disorders, PTSD, Eating Disorders, ODD) were similar in SA compared to PC, except ADHD, which was also more prevalent in the SA group. The medication profile was also similar across patient groups, but the SA group had significantly more individuals taking stimulants. The mean age for a first suicide attempt was 14.28 years old, ranging from 10 to 16 years old. The SA group presented a higher proportion of self-reported NSSI (87 vs. 64%). On average, the interval of time between the last suicide attempt and the clinical assessment was approximately 14 months (see Table 2).

### Self-reported impulsivity and suicidality

Differences across all 3 groups in impulsivity, measured with the BIS total score and on the attention subscale, were observed with the highest scores in SA and lowest in HC (*F* = 11.0, *p* < 0.0001). Impulsivity correlated strongly with suicidal ideation in the previous year as measured with SBQ-R (r = 0.49, *p* < 0.005), controlling for age, sex, and IQ.

### Symptoms characteristics of personality disorders, depressive disorders and suicidal behaviors

**Figure 1:**
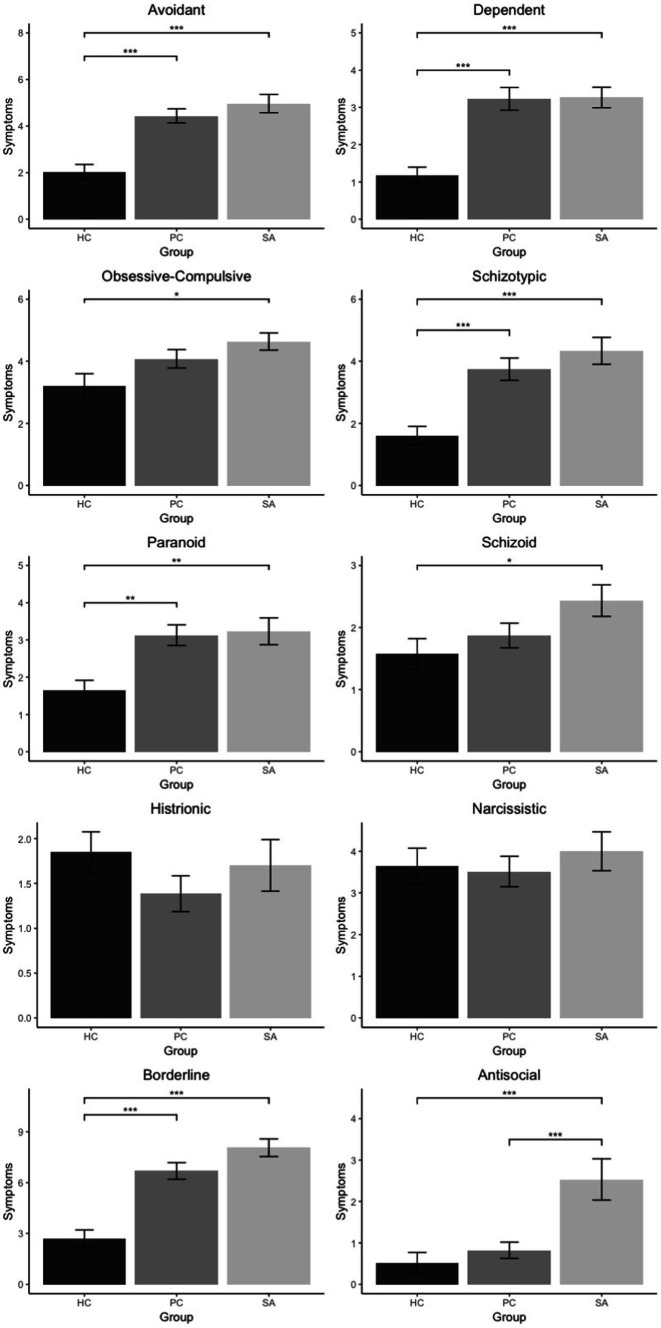
Level of symptoms characteristics of personality disorders across groups.

Groups presented numerous differences in SCPD scores measured with the SCID-II (see Figure 1). Certain scores of SCPD groups were more frequent across depressed adolescents (SA and PC) compared to HC, including avoidant group (*F* = 20.961, *p* < 0.001), dependent group (*F* = 18.537, *p* < 0.001), schizotypal group (*F* = 14.657, *p* < 0.001), paranoid group (*F* = 8.94, *p* < 0.001), and borderline traits (*F* = 30.03, *p* < 0.001). The only significant personality-related difference between SA and PC was found for symptoms characteristics of an antisocial personality disorder (*F* = 10.98, *p* < 0.001).

### Symptoms characteristics of personality disorders and suicidal ideation

**Figure 2:**
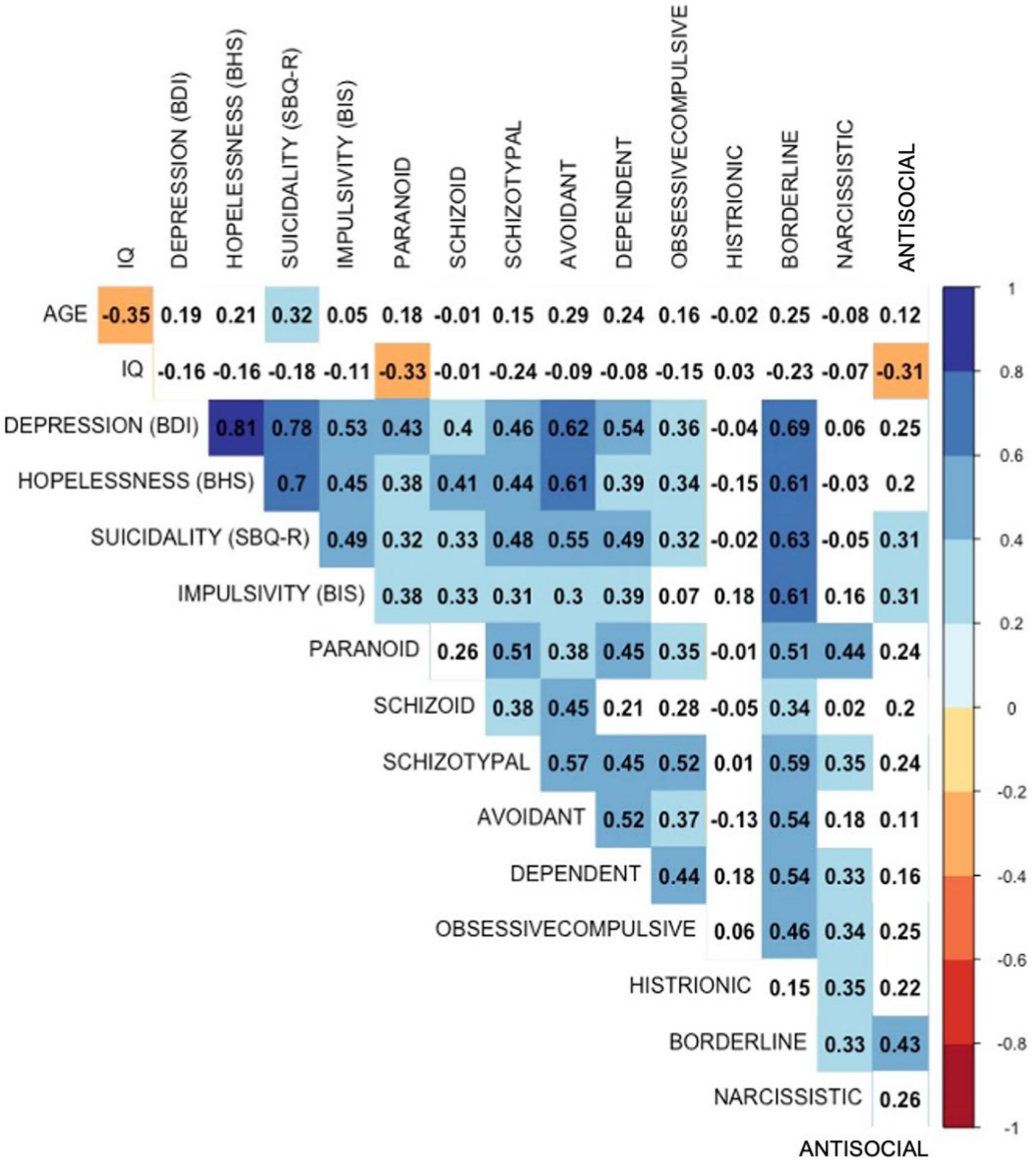
Correlational structure of groups of symptoms characteristics of personality disorders, age, impulsivity, depressive symptoms and suicidal ideation (SBQ-R score).

Cross-correlations between SCPD within diagnostic categories and suicidal ideation are presented in the correlation matrix (see Figure 2). Among all groups of SCPD categories, the borderline group was most strongly correlated with suicidal ideation (*r* = 0.69) and impulsivity (r = 0.62). Besides borderline, schizoid, avoidant, and dependent groups, also presented strong correlation with adolescent suicidal ideation (r’s around 0.5). Symptoms characteristics of an antisocial personality disorder presented a significant, yet smaller correlation with suicidal ideation.

### Specific symptoms characteristics of personality disorders and suicidal outcomes

Of all groups of SCPD examined with the SCID-II, a self-reported history of non-suicidal self-harm correlated most strongly with a history of SA (r_phi_ = −0.45) and score on the SBQ-R (r_pb_ = 0.69). Among the top 10 SCPD correlating with suicide attempt status, two other SCPD from the borderline group were present (chronic feeling of emptiness and behavioral impulsivity). The second most correlated SCPD with suicide attempt was a personal history of shoplifting or stealing (without confronting the victim). Lying or “conning” others were the second most correlated symptoms characteristic of an antisocial personality disorder, with suicide attempt (rank = 2, r_phi_ = 0.39). In contrast, the most correlated trait with suicidal ideation, were, respectively, symptoms characteristics of a borderline personality disorder (cut, burned, or scratched yourself) and an symptoms characteristics of an avoidant personality disorder (believing oneself as intellectually or physically inferior to others) (see Table 3).

**Table 3:** Correlational measures of specific symptoms characteristics of personality disorders (SCID-II) with past suicide attempt history and suicidal ideation (SBQ-R scores).

Correlational measure with suicide attempt history				
Rank	Criteria	Category	Statistics (r_phi_)	Confidence interval
1	Have you ever cut, burned, or scratched yourself on purpose?	Borderline	0.45	(0.16, 0.59)
2	Before you were 15, did you sometimes steal or shoplift things or forge someone’s signature?	Antisocial	0.39	(0.12, 0.54)
3	Do you often feel nervous when you are with other people?	Schizotypal	0.34	(0.09, 0.50)
4	Do you often feel empty inside?	Borderline	0.34	(0.09, 0.50)
5	Have you avoided jobs or tasks that involved having to deal with a lot of people?	Avoidant	0.33	(0.09, 0.49)
6	Do you believe that you are not as good, as smart, or as attractive as most other people	Avoidant	0.33	(0.09, 0.49)
7	Before you were 15, did you lie a lot or “con” other people?	Antisocial	0.33	(0.09, 0.49)
8	Have you often done things impulsively?	Borderline	0.33	(0.09, 0.49)
9	Do you often worry about being criticized or rejected in social situations?	Avoidant	0.32	(0.08, 0.48)
10	When you are out in public and see people talking, do you often feel that they are talking about you? “‘	Schizotypal	0.30	(0.07, 0.47)

Correlational measure with suicidal ideation				
Rank	Criteria	Category	Statistics (r_pb_)	Confidence interval (95%)
1	Have you ever cut, burned, or scratched yourself on purpose?	Borderline	0.69	0.30 0.78
2	Do you believe that you are not as good, as smart, or as attractive as most other people?	Avoidant	0.66	0.28 0.75
3	Do you often feel empty inside?	Borderline	0.65	0.27 0.75
4	Do you often worry about being criticized or rejected in social situations?	Avoidant	0.53	0.20 0.66
5	Do you often feel nervous when you are with other people?	Schizotypal	0.51	0.19 0.64
6	When you are out in public and see people talking, do you often feel that they are talking about you?	Schizotypal	0.49	0.18 0.62
7	Do you have lot of sudden mood changes?	Borderline	0.48	0.18 0.62
8	Are there really very few things that give you pleasure?	Schizoid	0.47	0.17 0.61
9	Do you worry a lot about being left alone to take care of yourself?	Dependent	0.46	0.16 0.60
10	Have you often done things impulsively?	Borderline	0.44	0.16 0.59

The top-10 items are presented for each suicidal outcome.

## Discussion

The objective of the current study was to identify SCPD that constitute risk factors for adolescent suicide attempts and suicidal ideation, beyond depressive disorders. While this study sought to examine SCPD in adolescents with depression and suicide in a controlled study, it is important to remember the fact that adolescent personality disorder traits or symptoms characteristics are still considered as typical developmental traits at this time of the adolescent life, rather than stable and sustained psychopathological traits or symptoms characteristics. Therefore, the following findings should be interpreted with caution and seen as proxy indicators only. After controlling for age, sex, and IQ differences, only one category of SCPD was specifically associated with suicide attempts in depressed adolescents: symptoms characteristic of an antisocial personality disorder. In addition, we found that self-reported impulsivity also correlated with suicide attempt history. Symptoms characteristics of various personality disorders – i.e. paranoid, schizotypal, avoidant, dependent, and borderline personality disorders – were more prevalent in all depressed adolescents (with or without suicide attempts) compared to healthy controls. In fact, the amount of SCPD correlated with suicidal ideation (except for histrionic and narcissistic personality disorders). Hence, many aspects of personality psychopathology appear to be risk factors for adolescent suicidal ideation, with impulsivity and antisocial tendencies showing a specific association with suicide attempts.

### Impulsivity and adolescent suicidal thoughts and behaviors

Several studies have previously identified impulsivity as a risk factor for suicidal behaviors in adults (47, 48) and adolescents (49). In the current study, the sub-dimension of attentional impulsivity correlated the most with suicide attempt history, in excess of subjects who fulfilled ADHD. Impulsivity did not only relate with suicide attempts but also displayed a close relationship with suicidal ideation. Likewise, Auerbach et al. found that certain sub-dimensions of impulsivity are more closely associated with adolescent suicide attempts (50). Using a different self-reported measure of impulsivity, the authors found that suicide attempts in adolescent inpatients were specifically associated with the tendency to react impulsively to emotional events. These findings are consistent with the current study, given the intricate relationship between affective and attentional systems in the brain (51). In addition, attentional impulsivity has been consistently associated with poor emotional regulation (52). Emotional dysregulation could mediate the observed relationship between impulsivity and adolescent suicide attempts (53). A history of childhood trauma might be an important moderator, as emotional reactivity was found to be associated with SA in adolescents with past sexual trauma, but not in adolescents without trauma (54). While emotional dysregulation could explain the relationship between suicidal ideation and impulsivity, the association between impulsivity and suicidal behaviors could be explained through poor inhibitory control (55). This is particularly relevant in the context adolescent development, a period characterized by ongoing cerebral maturation (56). Converging evidence suggests that impulsivity related to adolescent suicidal behaviors are associated with structural (57) and functional anomalies (58).

### Symptoms characteristic of an antisocial personality disorder and the vulnerability to suicidal behaviors in adolescents

The current study confirmed the relationship between antisocial tendencies and the risk for suicide attempts in adolescents. Previous studies have found a strong relatedness between adolescent suicide and antisocial behavior in 43% of suicide victims (59), which reflects that symptoms characteristics of an antisocial personality disorder might only characterize a subgroup of adolescent suicide attempters. Aggression, which is a construct related to antisociality (60), has also been associated with youth suicidal behaviors in a recent meta-analysis (61). The relationship between aggressive tendencies and suicidal behaviors has been found in adult (26) and in elderly samples (62), suggesting that this relationship occurs across the lifespan. Thus, the current study suggests that the impulsive-aggressive phenotype associated with suicide risk emerges before adulthood. Indeed, both impulsivity and the tendency for aggression are traits that emerge early in development, with individual differences present even before puberty (63).

The combined results of increased impulsivity and antisocial behaviors found in adolescent suicide attempters support the role of impulsive-aggression in suicide risk (64). The developmental cascade that links impulsive-aggression and suicide attempts is complex and several biological systems have been implicated (65). The impulsive-aggressive phenotype has been associated with the familial heritability of suicide. Dysregulation of serotonin systems has been linked to disruptive behaviors in youth (66) and has been found to play a role in mediating the relationship between impulsive-aggressive personality traits and suicidal behaviors, particularly in relation to early life adversity (67). Furthermore, well-characterized epigenetic risk factors for suicide, such as hyper-methylated promotor of the hippocampal glucocorticoid receptor, also impact suicide risk (at least in part) through enduring trait-like personality features (68). In sum, continuing to expand our knowledge of personality vulnerability factors in suicidal risk remains important given the implication of personality traits in mediating the biological risk factors of suicide (69).

Notably our sample was limited in size and biased toward female adolescents, which reflected the clinical population of the depressive disorder clinic where they were recruited. Typically, violent and delinquent behaviors are more prevalent in male adolescents than in females, leading to increased rates of symptoms characteristic of an antisocial personality disorder in male adolescents. However, the analysis was controlled for sex, suggesting that the association between aggressive behaviors and suicidal history was not driven by an over-representation of males. In fact, the SA groups contained fewer males than the two other groups. Examining how sex moderates the relationship between suicidal and antisocial behaviors warrant further study.

Adolescents in the SA group exhibited elevated impulsivity scores on the Barrat Impulsiveness Scale, received more frequent diagnoses of ADHD, and consequently, a higher frequency of stimulant medication prescription, which are widely acknowledged as independant risk factors for suicide attempts (70). Of note, adolescents in the SA group could have a high impulsivity score without fulfilling the diagnostic criteria of ADHD. Also, while no statistically significant difference was detected with respect to substance use disorder, it is important to mention that these findings should be interpreted with caution, given the recruitment process of the patients at the Depressive Disorders Clinic for adolescents. Adolescents included in our study could have used substances without fulfilling criteria for a substance use disorder. Adolescents fulfilling diagnostic criterai for a substance use disorder are first triaged and then referred to addiction services, thereby decreasing the representation of substance us disorder in our sample. Previous studies described how motor impulsivity might increase the risk of suicide attempt in patients with substance use disorders (71).

### Beyond the impulsive-aggressive phenotype: symptoms characteristics of personality disorders and suicidal ideation

In the current analysis, most categories of SCPD were associated with suicidal ideation, highlighting the wide heterogeneity of mechanistic pathways that can lead to suicidal ideation. Borderline personality symptoms appeared to play a central role in the clinical presentation of this population. While symptoms characteristics of a borderline personality disorder did not distinguish depressed adolescents with past suicide attempts from those without, these traits showed the highest correlation with depressive symptoms, hopelessness, impulsivity, antisocial personality traits and suicidal ideation. The single items that correlated the most with suicide attempts and suicidal ideation was the presence of NSSI. These results replicate the well-established association between non-suicidal self-injury and adolescent suicidality (69). Interestingly, the second borderline item presenting the highest correlation with adolescent suicidality was a chronic feeling of emptiness. Feeling of emptiness has been associated with feelings of isolation, loneliness, and hopelessness (24). In addition to symptoms characteristics of a borderline personality disorder, many personality items related to social anxiety and low self-esteem presented high correlations with suicidal ideation. Taken together, these results indicate that clinical and research attention should be given to feeling of emptiness and social anxiety in the assessment of adolescent suicidal diathesis.

#### Limitation of the findings

SCPD observed in this sample of adolescents with depression and suicidal behaviors should be interpreted with caution; while differences in the number of symptoms characteristics be seen as normal and transitent developmental state. As mentionned above, the sample size of each group limits the significance of the conlusions, as well as the different time frames between these studied variables between SCPD and lifetime suicide risk. Therefore, further methodological and design refinements need to be considered.

### Clinical implications and future directions

The current study indicates that examining SCPD is highly relevant for characterizing depressed adolescents at high-risk of suicide. The current study strengthens the view that impulsivity and antisocial behaviors are important risk factors for suicide attempts during adolescence. This observation is important for early detection and clinical intervention aiming to prevent suicide. Psychotherapeutic interventions targeting emerging SCPD in adolescents may, in combination with treatment for depression, help reduce suicidal risk. The current study also highlights the differential contribution of SCPD on suicidal thoughts and behaviors. In addition to NSSI, the current study showed potential associations of suicide attempts with specific symptoms characteristics of antisocial personality disorder/conduct disorder symptoms (shoplifting/lying), which typically do not receive much clinical attention.

Limitations of the current study include a cross-sectional design, which limits any causal interpretation. Prognostic interpretations should always be considered regarding the instability of personality disorders in adolescence (72). Longitudinal studies are needed to establish the predictive role of personality features for suicide risk (14). Future studies should also evaluate if these emerging personality traits are amenable to pharmacological or psychosocial treatment. Dialectical bahavior therapy intervention, which has shown promising results in decreasing adolescent suicide risk (32), already targets reckless behaviors, emotional dysregulation and aggression (73). Future interventional trials could establish the most malleable targets to personalize treatment.

## Conclusion

Adolescence is a critical developmental period where suicidal thoughts and behaviors, mood disorders and pathological personality traits emerge. Understanding the mechanisms underlying the emergence of suicidal behaviors during this period is key to developing improved preventive and therapeutic strategies. Girls with antisocial behavior require special vigilance when it comes to depressive episodes. While impulsive aggressive phenotypes seem to underlie adolescent suicide attempt risk, a careful characterization of personality psychopathology appears important to a person-centered psychotherapy in depressed adolescents.

## Data availability statement

The original contributions presented in the study are included in the article/supplementary material, further inquiries can be directed to the corresponding author.

## Ethics statement

The studies involving humans were approved by Douglas Institute Research Ethics Board. The studies were conducted in accordance with the local legislation and institutional requirements. Written informed consent for participation in this study was provided by the participants' legal guardians/next of kin.

## Author contributions

AG: Formal analysis, Validation, Writing – original draft, Software. MS: Validation, Writing – review & editing. GL: Software, Formal analysis, Writing – review & editing. SM: Writing – review & editing. FJ: Conceptualization, Project administration, Writing – review & editing. JR: Conceptualization, Project administration, Writing – review & editing, Data curation, Formal analysis, Funding acquisition, Investigation, Methodology, Resources, Supervision, Validation, Writing – original draft.

## References

[1] Cash SJ Bridge JA. Epidemiology of youth suicide and suicidal behavior. Curr Opin Pediatr. (2009) 21:613–9. doi: 10.1097/MOP.0b013e32833063e1, PMID: 19644372 PMC2885157

[2] Nock MK Green JG Hwang I McLaughlin KA Sampson NA Zaslavsky AM. Prevalence, correlates, and treatment of lifetime suicidal behavior among adolescents: results from the National Comorbidity Survey Replication Adolescent Supplement. JAMA Psychiatry. (2013) 70:300–10. doi: 10.1001/2013.jamapsychiatry.55, PMID: 23303463 PMC3886236

[3] World Health Organization Suicide worldwide in 2019: Global Health estimates [internet]. (2021). Available at: https://www.who.int/publications-detail-redirect/9789240026643

[4] Mokdad AH Forouzanfar MH Daoud F Mokdad AA El Bcheraoui C Moradi-Lakeh M. Global burden of diseases, injuries, and risk factors for young people’s health during 1990–2013: a systematic analysis for the global burden of disease study 2013. Lancet. (2016) 387:2383–401. doi: 10.1016/S0140-6736(16)00648-6, PMID: 27174305

[5] Cha CB Franz PJ Guzmán E Glenn CR Kleiman EM Nock MK. Annual research review: suicide among youth - epidemiology, (potential) etiology, and treatment. J Child Psychol Psychiatry. (2017) 59:460–82. doi: 10.1111/jcpp.12831, PMID: 29090457 PMC5867204

[6] Bilsen J. Suicide and youth: Risk factors. Front Psychiatry. (2018) 9:540. doi: 10.3389/fpsyt.2018.00540 30425663 PMC6218408

[7] Fergusson DM Woodward LJ Horwood LJ. Risk factors and life processes associated with the onset of suicidal behaviour during adolescence and early adulthood. Psychol Med. (2000) 30:23–39. doi: 10.1017/S003329179900135X, PMID: 10722173

[8] Conwell Y Duberstein PR Cox C Herrmann JH Forbes NT Caine ED. Relationships of age and axis I diagnoses in victims of completed suicide: a psychological autopsy study. Am J Psychiatry. (1996) 153:1001–8. doi: 10.1176/ajp.153.8.1001 8678167

[9] Cavanagh JTO Carson AJ Sharpe M Lawrie SM. Psychological autopsy studies of suicide: a systematic review. Psychol Med. (2003) 33:395–405. doi: 10.1017/S0033291702006943, PMID: 12701661

[10] McGirr A Renaud J Bureau A Seguin M Lesage A Turecki G. Impulsive-aggressive behaviours and completed suicide across the life cycle: a predisposition for younger age of suicide. Psychol Med. (2008) 38:407–17. doi: 10.1017/S0033291707001419, PMID: 17803833

[11] Franklin JC Ribeiro JD Fox KR Bentley KH Kleiman EM Huang X. Risk factors for suicidal thoughts and behaviors: a meta-analysis of 50 years of research. Psychol Bull. (2017) 143:187–232. doi: 10.1037/bul0000084 27841450

[12] Mann JJ Waternaux C Haas GL Malone KM. Toward a clinical model of suicidal behavior in psychiatric patients. Am J Psychiatry. (1999) 156:181–9. doi: 10.1176/ajp.156.2.181, PMID: 9989552

[13] Brezo J Paris J Turecki G. Personality traits as correlates of suicidal ideation, suicide attempts, and suicide completions: a systematic review. Acta Psychiatr Scand. (2006) 113:180–206. doi: 10.1111/j.1600-0447.2005.00702.x, PMID: 16466403

[14] Yen S Shea MT Sanislow CA Skodol AE Grilo CM Edelen MO. Personality traits as prospective predictors of suicide attempts. Acta Psychiatr Scand. (2009) 120:222–9. doi: 10.1111/j.1600-0447.2009.01366.x, PMID: 19298413 PMC2729360

[15] O’Connor RC. The relations between perfectionism and suicidality: a systematic review. Suicide Life Threat Behav. (2007) 37:698–714. doi: 10.1521/suli.2007.37.6.698, PMID: 18275376

[16] Batterham PJ Christensen H. Longitudinal risk profiling for suicidal thoughts and behaviours in a community cohort using decision trees. J Affect Disord. (2012) 142:306–14. doi: 10.1016/j.jad.2012.05.021, PMID: 22840465

[17] Blüml V Kapusta ND Doering S Brähler E Wagner B Kersting A. Personality factors and suicide risk in a representative sample of the German general population. PLoS One. (2013) 8:e76646. doi: 10.1371/journal.pone.0076646, PMID: 24124582 PMC3790756

[18] Batty GD Gale CR Tanji F Gunnell D Kivimäki M Tsuji I. Personality traits and risk of suicide mortality: findings from a multi-cohort study in the general population. World Psychiatry. (2018) 17:371–2. doi: 10.1002/wps.20575 30229569 PMC6127810

[19] Roxborough HM Hewitt PL Kaldas J Flett GL Caelian CM Sherry S. Perfectionistic self-presentation, socially prescribed perfectionism, and suicide in youth: a test of the perfectionism social disconnection model: perfectionism, social disconnection, and suicide. Suicide Life Threat Behav. (2012) 42:217–33. doi: 10.1111/j.1943-278X.2012.00084.x, PMID: 22380005

[20] Gorlyn M. Impulsivity in the prediction of suicidal behavior in adolescent population. Int J Adolesc Med Health. (2005) 17. doi: 10.1515/ijamh.2005.17.3.205 16231471

[21] Bagge CL Littlefield AK Rosellini AJ Coffey SF. Relations among behavioral and questionnaire measures of impulsivity in a sample of suicide attempters. Suicide Life Threat Behav. (2013) 43:460–7. doi: 10.1111/sltb.12030, PMID: 23601164 PMC4618602

[22] May AM Klonsky ED. What distinguishes suicide attempters from suicide ideators? A meta-analysis of potential factors. Clin Psychol Sci Pract. (2016) 23:5–20. doi: 10.1111/cpsp.12136

[23] Klonsky ED Saffer BY Bryan CJ. Ideation-to-action theories of suicide: a conceptual and empirical update. Curr Opin Psychol. (2018) 22:38–43. doi: 10.1016/j.copsyc.2017.07.020, PMID: 30122276

[24] Giegling I Olgiati P Hartmann AM Calati R Möller HJ Rujescu D. Personality and attempted suicide. Analysis of anger, aggression and impulsivity. J Psychiat Res. (2009) 43:1262–71. doi: 10.1016/j.jpsychires.2009.04.013, PMID: 19481222

[25] Gvion Y Apter A. Aggression, impulsivity, and suicide behavior: a review of the literature. Arch Suicide Res. (2011) 15:93–112. doi: 10.1080/13811118.2011.565265, PMID: 21541857

[26] Dumais A Lesage AD Lalovic A Séguin M Tousignant M Chawky N. Is violent method of suicide a behavioral marker of lifetime aggression? Am J Psychiatry. (2005) 162:1375–8. doi: 10.1176/appi.ajp.162.7.1375, PMID: 15994723

[27] Sharp C Vanwoerden S Wall K. Adolescence as a sensitive period for the development of personality disorder. Psychiatr Clin North Am. (2018) 41:669–83. doi: 10.1016/j.psc.2018.07.004, PMID: 30447731

[28] Meijer M Goedhart AW PDA T. The persistence of borderline personality disorder in adolescence. J Personal Disord. (1998) 12:13–22. doi: 10.1521/pedi.1998.12.1.13, PMID: 9573516

[29] Krueger RF Hicks BM Patrick CJ Carlson SR Iacono WG McGue M. Etiologic connections among substance dependence, antisocial behavior and personality: modeling the externalizing spectrum. J Abnorm Psychol. (2002) 111:411–24. doi: 10.1037/0021-843X.111.3.411, PMID: 12150417

[30] Kotov R Gamez W Schmidt F Watson D. Linking “big” personality traits to anxiety, depressive, and substance use disorders: a meta-analysis. Psychol Bull. (2010) 136:768–821. doi: 10.1037/a0020327 20804236

[31] McCauley E Berk MS Asarnow JR Adrian M Cohen J Korslund K. Efficacy of dialectical behavior therapy for adolescents at high risk for suicide: a randomized clinical trial. JAMA Psychiatry. (2018) 75:777–85. doi: 10.1001/jamapsychiatry.2018.1109 29926087 PMC6584278

[32] Sharp C. Bridging the gap: the assessment and treatment of adolescent personality disorder in routine clinical care. Arch Dis Child. (2017) 102:103–8. doi: 10.1136/archdischild-2015-310072, PMID: 27507846

[33] Posner K Oquendo MA Gould M Stanley B Kopper BA Davies M. Columbia Classification Algorithm of Suicide Assessment (C-CASA): classification of suicidal events in the FDA’s pediatric suicidal risk analysis of antidepressants. Am J Psychiatry.. (2007) 164:1035–43. doi: 10.1176/ajp.2007.164.7.1035 17606655 PMC3804920

[34] Gifuni AJ Chakravarty MM Lepage M Ho TC Geoffroy MC Lacourse E. Brain cortical and subcortical morphology in adolescents with depression and a history of suicide attempt. J Psychiatry Neurosci. (2021) 46:E347–57. doi: 10.1503/jpn.200198, PMID: 33961355 PMC8327980

[35] Brent DA Oquendo M Birmaher B Greenhill L Kolko D Stanley B. Familial pathways to early-onset suicide attempt. Arch Gen Psychiatry. (2002) 59:801–7. doi: 10.1001/archpsyc.59.9.801, PMID: 12215079

[36] Jollant F Wagner G Richard-Devantoy S Köhler S Bär KJ Turecki G. Neuroimaging-informed phenotypes of suicidal behavior: a family history of suicide and the use of a violent suicidal means. Transl Psychiatry. (2018) 8:120. doi: 10.1038/s41398-018-0170-2, PMID: 29921964 PMC6008434

[37] Kaufman J Birmaher B Brent D Rao U Flynn C Moreci P. Schedule for affective disorders and schizophrenia for school-age children-present and lifetime version (K-SADS-PL): initial reliability and validity data. J Am Acad Child Adolesc Psychiatry. (1997) 36:980–8. doi: 10.1097/00004583-199707000-00021, PMID: 9204677

[38] Osman A Bagge CL Gutierrez PM Konick LC Kopper BA Barrios FX. The suicidal behaviors questionnaire-revised (SBQ-R): validation with clinical and nonclinical samples. Assessment. (2001) 8:443–54. doi: 10.1177/107319110100800409, PMID: 11785588

[39] First MB Gibbon M. The structured clinical interview for DSM-IV axis I disorders (SCID-I) and the structured clinical interview for DSM-IV axis II disorders (SCID-II). (2004). American Psychiatric Press, Inc.

[40] Patton JH Stanford MS Barratt ES. Factor structure of the barratt impulsiveness scale. J Clin Psychol. (1995) 51:768–74. doi: 10.1002/1097-4679(199511)51:6<768::AID-JCLP2270510607>3.0.CO;2-1 8778124

[41] Barrera M Garrison-Jones CV. Properties of the beck depression inventory as a screening instrument for adolescent depression. J Abnorm Child Psychol. (1988) 16:263–73. doi: 10.1007/BF00913799 3403810

[42] Beck AT Weissman A Lester D Trexler L. The measurement of pessimism: the hopelessness scale. J Consult Clin Psychol. (1974) 42:861–5. doi: 10.1037/h0037562, PMID: 4436473

[43] Granö N Oksanen J Kallionpää S Roine M. Specificity and sensitivity of the Beck hopelessness scale for suicidal ideation among adolescents entering early intervention service. Nord J Psychiatry. (2017) 71:72–6. doi: 10.1080/08039488.2016.1227370, PMID: 27626513

[44] Wechsler D. Wechsler intelligence scale for children, fourth edition [internet] American Psychological Association (2012) Available at: http://doi.apa.org/getdoi.cfm?doi=10.1037/t15174-000.

[45] Kaufman AS. Test review: Wechsler, D. Manual for the Wechsler adult intelligence scale, revised: New York: Psychological Corporation, 1981. J Psychoeduc Assess. (1983) 1:309–13.

[46] Brodsky BS Oquendo M Ellis SP Haas GL Malone KM Mann JJ. The relationship of childhood abuse to impulsivity and suicidal behavior in adults with major depression. Am J Psychiatry. (2001) 158:1871–7. doi: 10.1176/appi.ajp.158.11.1871, PMID: 11691694

[47] Dal Santo F Carballo JJ Velasco A Jiménez-Treviño L Rodríguez-Revuelta J Martínez-Cao C. The mediating role of impulsivity in the relationship between suicidal behavior and early traumatic experiences in depressed subjects. Front Psych. (2020) 11:538172. doi: 10.3389/fpsyt.2020.538172, PMID: 33240115 PMC7683571

[48] McHugh CM Lee RSC Hermens DF Corderoy A Large M Hickie IB. Impulsivity in the self-harm and suicidal behavior of young people: a systematic review and meta-analysis. J Psychiatr Res. (2019) 116:51–60. doi: 10.1016/j.jpsychires.2019.05.012, PMID: 31195164

[49] Auerbach RP Stewart JG Johnson SL. Impulsivity and suicidality in adolescent inpatients. J Abnorm Child Psychol. (2017) 45:91–103. doi: 10.1007/s10802-016-0146-8, PMID: 27025937 PMC5045310

[50] Sander D Grandjean D Pourtois G Schwartz S Seghier ML Scherer KR. Emotion and attention interactions in social cognition: brain regions involved in processing anger prosody. NeuroImage. (2005) 28:848–58. doi: 10.1016/j.neuroimage.2005.06.023 16055351

[51] Garofalo C Velotti P Zavattini GC. Emotion regulation and aggression: the incremental contribution of alexithymia, impulsivity, and emotion dysregulation facets. Psychol Violence. (2018) 8:470–83. doi: 10.1037/vio0000141

[52] Ammerman BA Kleiman EM Uyeji LL Knorr AC McCloskey MS. Suicidal and violent behavior: the role of anger, emotion dysregulation, and impulsivity. Personal Individ Differ. (2015) 79:57–62. doi: 10.1016/j.paid.2015.01.044

[53] Stewart JG Kim JC Esposito EC Gold J Nock MK Auerbach RP. Predicting suicide attempts in depressed adolescents: clarifying the role of disinhibition and childhood sexual abuse. J Affect Disord. (2015) 187:27–34. doi: 10.1016/j.jad.2015.08.034, PMID: 26318268 PMC4587293

[54] Fino E Melogno S Iliceto P D’Aliesio S Pinto MA Candilera G. Executive functions, impulsivity, and inhibitory control in adolescents: a structural equation model. Adv Cogn Psychol. (2014) 10:32–8. doi: 10.5709/acp-0154-5, PMID: 25157298 PMC4118776

[55] Guyer AE Silk JS Nelson EE. The neurobiology of the emotional adolescent: from the inside out. Neurosci Biobehav Rev. (2016) 70:74–85. doi: 10.1016/j.neubiorev.2016.07.037, PMID: 27506384 PMC5074886

[56] Fradkin Y Khadka S Bessette KL Stevens MC. The relationship of impulsivity and cortical thickness in depressed and non-depressed adolescents. Brain Imaging Behav. (2017) 11:1515–25. doi: 10.1007/s11682-016-9612-8, PMID: 27738995

[57] Gifuni AJ Perret LC Perret E Geoffroy MC Mbekou V Jollant F Renaud J. Decision-making and cognitive control in adolescent suicidal behaviors: a qualitative systematic review of the literature. European Child & Adolescent Psychiatry. (2020) 9:1–7. 10.1007/s00787-020-01550-3 32388626

[58] Marttunen MJ Aro HM Henriksson MM Lönnqvist JK. Antisocial behaviour in adolescent suicide. Acta Psychiatr Scand. (1994) 89:167–73. doi: 10.1111/j.1600-0447.1994.tb08087.x 8178674

[59] Coie JD Dodge KA. Aggression and antisocial behavior. (1998). John Wiley & Sons, Inc.

[60] Hartley CM Pettit JW Castellanos D. Reactive aggression and suicide-related behaviors in children and adolescents: a review and preliminary meta-analysis. Suicide Life-Threat Behav. (2018) 48:38–51. doi: 10.1111/sltb.12325, PMID: 28044358 PMC7894982

[61] Szücs A Szanto K Aubry JM Dombrovski AY. Personality and suicidal behavior in old age: a systematic literature review. Front Psych. (2018) 9:128. doi: 10.3389/fpsyt.2018.00128, PMID: 29867594 PMC5949532

[62] Frick PJ White SF. Research review: the importance of callous-unemotional traits for developmental models of aggressive and antisocial behavior. J Child Psychol Psychiatry. (2008) 49:359–75. doi: 10.1111/j.1469-7610.2007.01862.x, PMID: 18221345

[63] Renaud J Berlim MT McGirr A Tousignant M Turecki G. Current psychiatric morbidity, aggression/impulsivity, and personality dimensions in child and adolescent suicide: a case-control study. J Affect Disord. (2008) 105:221–8. doi: 10.1016/j.jad.2007.05.013, PMID: 17568682

[64] Turecki G Ernst C Jollant F Labonté B Mechawar N. The neurodevelopmental origins of suicidal behavior. Trends Neurosci. (2012) 35:14–23. doi: 10.1016/j.tins.2011.11.008, PMID: 22177979

[65] Berman ME Tracy JI Coccaro EF. The serotonin hypothesis of aggression revisited. Clin Psychol Rev. (1997) 17:651–65. doi: 10.1016/S0272-7358(97)00039-1 9336689

[66] Lopez-Castroman J Jaussent I Beziat S Guillaume S Baca-Garcia E Genty C. Increased severity of suicidal behavior in impulsive aggressive patients exposed to familial adversities. Psychol Med. (2014) 44:3059–68. doi: 10.1017/S0033291714000646, PMID: 25065374

[67] Lutz PE Mechawar N Turecki G. Neuropathology of suicide: recent findings and future directions. Mol Psychiatry. (2017) 22:1395–412. doi: 10.1038/mp.2017.141, PMID: 28696430

[68] Beauchaine TP Zisner AR Sauder CL. Trait impulsivity and the externalizing Spectrum. Annu Rev Clin Psychol. (2017) 13:343–68. doi: 10.1146/annurev-clinpsy-021815-093253 28375718

[69] Glenn CR Lanzillo EC Esposito EC Santee AC Nock MK Auerbach RP. Examining the course of suicidal and nonsuicidal self-injurious thoughts and behaviors in outpatient and inpatient adolescents. J Abnorm Child Psychol. (2017) 45:971–83. doi: 10.1007/s10802-016-0214-0, PMID: 27761783 PMC5397367

[70] Geoffroy MC Orri M Girard A Perret LC Turecki G. Trajectories of suicide attempts from early adolescence to emerging adulthood: prospective 11-year follow-up of a Canadian cohort. Psychological medicine.. (2021) 51:1933–43. 32290876 10.1017/S0033291720000732

[71] Rodríguez-Cintas L Daigre C Braquehais MD Palma-Alvarez RF Grau-López L Ros-Cucurull E. Factors associated with lifetime suicidal ideation and suicide attempts in outpatients with substance use disorders. Psychiatry Research.. (2018) 1:262–440-5. 10.1016/j.psychres.2017.09.021 28951146

[72] Glenn CR Lanzillo EC Esposito EC Santee AC Nock MK Auerbach RP. Examining the course of suicidal and nonsuicidal self-injurious thoughts and behaviors in outpatient and inpatient adolescents. J Abnorm Child Psychol.. (2017) 45:971–83. doi: 10.1007/s10802-016-0214-0 27761783 PMC5397367

[73] Wilks CR Korslund KE Harned MS Linehan MM. Dialectical behavior therapy and domains of functioning over two years. Behav Res Ther. (2016) 77:162–9. doi: 10.1016/j.brat.2015.12.013, PMID: 26764586 PMC4752852

